# Self-Nucleation Enables Polymorphic Selection in Thermoplastic Polyurethanes

**DOI:** 10.1021/acs.macromol.5c01477

**Published:** 2025-09-12

**Authors:** Zakarya Baouch, Leire Sangroniz, Yunxiang Shi, Elmar Pöselt, Alejandro J. Müller, Dario Cavallo

**Affiliations:** † Department of Chemistry and Industrial Chemistry, 9302 University of Genoa, Via Dodecaneso 31, 16146 Genoa, Italy; ‡ POLYMAT and Department of Polymers and Advanced Materials: Physics, Chemistry and Technology, Faculty of Chemistry, 160665 University of the Basque Country UPV/EHU, Paseo Manuel de Lardizábal, 3, Donostia-San Sebastián 20018, Spain; § BASF Polyurethanes GmbH, A30, Elastogranstraße 60, 49448 Lemförde, Germany; ∥ IKERBASQUE, Basque Foundation for Science, Plaza Euskadi 5, 48009 Bilbao, Spain

## Abstract

This work investigates the self-nucleation behavior of thermoplastic polyurethanes (TPUs) with hard segment (HS) contents ranging from 29 to 80 wt %. Differential scanning calorimetry (DSC) reveals that upon cooling from the isotropic melt (*Domain I*), crystallization initially occurs as a single low-temperature exothermic peak associated with the formation of metastable *Form I*. However, when the self-nucleation temperature (*T*_s_) is within *Domain II* (the self-nucleation *Domain*), a second, higher-temperature crystallization exotherm emerges and progressively dominates as *T*_s_ decreases, indicating a change in polymorphic crystallization to the more ordered *Form II*. Therefore, self-nucleation not only accelerates crystallization kinetics but also alters the polymorphic outcome, favoring *Form II* over *Form I*. This interpretation is further supported by ex situ Wide-Angle X-ray Diffraction (WAXD) and polarized light optical microscopy (PLOM) measurements, which confirm the increasing presence of *Form II* with decreasing *T*_s_, as evidenced by its characteristic diffraction patterns and by the growing presence of *Form II* birefringent spherulites, particularly in high-HS-content TPUs. Notably, even TPUs with low HS content (29–33%), which are typically incapable of crystallizing in *Form II* under nonisothermal conditions, develop this polymorph induced by the thermal treatment applied by self-nucleation. The reason behind the formation of *Form II* by self-nucleation is the persistence of interurethane hydrogen bonds in the melt, which may favor the crystallization of *Form II* due to its higher content of bonded carbonyl and N–H groups with respect to *Form I*. These findings demonstrate that self-nucleation enables precise control over polymorphic selection in TPUs across a wide compositional range, offering a versatile strategy for tailoring material properties through thermal processing.

## Graphical abstract

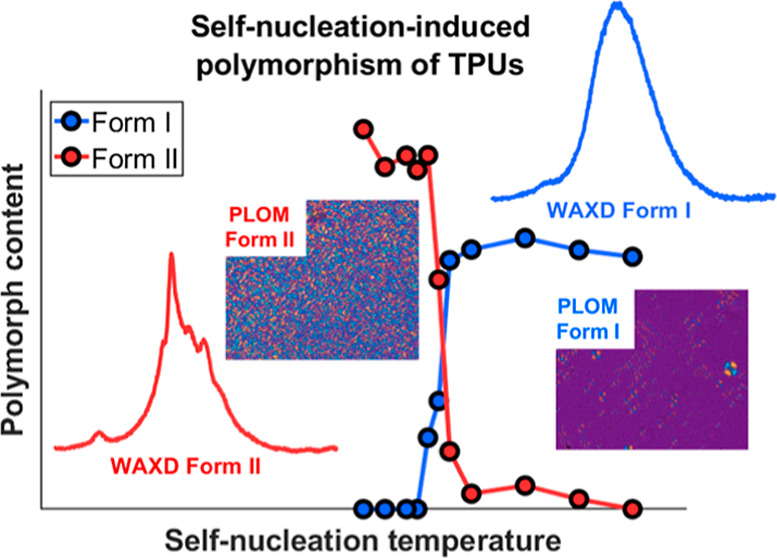

## Introduction

Thermoplastic polyurethanes (TPUs) are segmented multiblock copolymers consisting of hard and soft segments (HS and SS, respectively) within which urethane groups link the blocks.^1,2^ By tailoring the hard and soft segment content, a wide range of mechanical properties can be achieved, which leads to diverse applications from footwear to smart materials.^3,4^ From a chemical point of view, the most common linear polyurethanes are formed by the reaction of a diisocyanate with a short diol (forming the HS) and a chain extended with a short polyester or polyether (producing the SS). A particularly relevant class of TPUs is the one based on 4,4′-methylenediphenyl di-isocyanate/1,4-butanediol (MDI/BDO) as HS and poly­(tetramethylene oxide) (PTMO) as SS. The HS of these materials can easily crystallize from the melt, driven by the hydrogen bonding interactions among the urethane groups.^5^

Interestingly, different crystal polymorphs can develop, mainly depending on solidification conditions.^6–9^ In particular, the most thermodynamically stable crystal form is the more ordered *Form II*, characterized by a triclinic lattice^7,9,10^ and crystallizing at higher temperatures.^11–13^ On the other hand, at larger undercoolings, the more disordered and paracrystalline *Form I* emerges.^11–13^ Recently, we investigated in detail the effect of the cooling rate from the melt on the polymorphism of TPUs with different contents of hard segments.^14^ We showed that *Form I* crystallizes exclusively independently of cooling rate for HS content lower than 50 wt % and dominates as a crystallization product at cooling rates larger than approximately 20 °C/min for larger HS percentages. Lower cooling rates lead to a mixture of the two polymorphs in the solid state, with the quantity of *Form II* increasing with a decreasing cooling rate. Pure *Form II* cannot be achieved due to the concurrent thermal degradation of the TPUs at cooling rates lower than 1–3 °C/min.^14^

As such, thermal history is of primary importance to control TPUs polymorphism. A peculiar thermal protocol applied to polymer crystallization studies by DSC is self-nucleation.^15–18^ First, the material is melted at a high enough temperature to erase all thermal history. Then, the material is cooled at a constant scanning rate to produce a “standard semicrystalline state”. Next, the sample is heated to a temperature denoted as *T*_s_ (for self-nucleation temperature), where it remains for a constant time (typically 3 or 5 min), after which the sample is cooled again at the same rate to observe the effects of the thermal treatment on the nonisothermal crystallization of the material. The final step is a heating scan to melt all crystals produced during this thermal protocol.^15–18^ When *T*_s_ is high enough, no effect of the melting treatment on recrystallization can be observed, and the material is in the so-called *Domain I* (i.e., the isotropic melting *Domain*). At self-nucleation temperatures lower than a critical value, the nonisothermal crystallization kinetics become accelerated, as demonstrated by increasing crystallization temperatures, and the polymer enters *Domain II* or the self-nucleation *Domain* (where the material is self-nucleated without any annealing effects, in the case crystal fragments remain in the melt). At even lower *T*_s_ temperatures, the material is only partially melted; thus, the surviving crystals anneal (i.e., thicken), and the sample enters the Self-Nucleation and Annealing *Domain* or *Domain III*. Other than the recrystallization kinetics, self-nucleation can also determine which of the possible crystalline polymorphs will develop in polymorphic polymers.^19–30^

Concerning TPUs, in previous studies, self-nucleation has been applied to polymers with HS content ranging from 30 to 43 wt %. It was demonstrated that the obtained relevant increase in the nonisothermal crystallization temperature with self-nucleation led to a tailoring of the melting point, which increased up to about 20 °C.^31^ Moreover, a substantial increase in the nucleation density and the development of thicker crystalline lamellae were also reported.^31^ The self-nucleation protocol has also been used to define an efficiency scale for TPUs nucleating agents^32^ and as a first step to thermally fractionate the segmented multiblock copolymers’ crystallizable sequences.^33^

However, previous works on self-nucleation of TPUs have not studied an extended range of polymer composition (HS content) or focused on self-nucleation’s effects on polymorphic crystallization (*Form II/Form I* development). The present work combines differential scanning calorimetry measurements with structural and morphological evaluations to fill this knowledge gap.

## Materials

The TPUs used in this study were synthesized by BASF Polyurethanes GmbH (Lemförde, Germany) via a one-shot polymerization process. These polymers are based on 4,4′-methylenediphenyl diisocyanate (MDI) and 1,4-butanediol (BD), which together constitute the hard segment (HS) components. The soft segments (SS) are derived from poly­(tetramethylene oxide)­macrodiol, featuring a number-average molecular weight (*M*_n_) of approximately 1000 g/mol and a polydispersity index of approximately 2. The NCO index (OH/NCO ratio) of the TPU synthesis was 990, meaning that a slightly higher amount of OH groups was employed to avoid the formation of reversible cross-links such as allophanates.

Following synthesis, TPU was ground into small chips and subsequently processed through injection molding to form standardized test sheets. Before characterization, the samples underwent an annealing treatment at 100 °C for 20 h to promote phase separation between hard and soft segments. Table 1 details the composition and key characteristics of the TPU specimens evaluated in this research. The MDI-BD length and the molar masses were measured via ^13^C NMR and GPC, respectively, as reported in a previous publication.^14^

**Table 1:** Composition and Properties of the Studied TPUs

sample code	HS content [%]	MDI-BD avg. length [repeating unit]	*M*_w_ [g/mol]	*M*_n_ [g/mol]	polydispersity index (PI)
TPU29	29.6	2.2	99,000	45,000	2.2
TPU33	33.2	2.3	101,000	45,000	2.2
TPU50	50.0	3.7	98,000	43,000	2.3
TPU60	60.0	5.0	97,000	43,000	2.3
TPU70	70.0	7.6	93,000	41,000	2.3
TPU80	80.0	10.8	83,000	37,000	2.2

## Instrumentation

### Differential Scanning Calorimetry (DSC)

DSC analyses were performed using a DSC250 instrument (TA Instruments, Newcastle, Delaware, USA) under a nitrogen atmosphere with a flow rate of 50 mL/min. Temperature and heat flow signals were calibrated by using a high-purity indium reference. For each test, a new sample of around 8 mg was used to avoid any effects of prior thermal exposure (and possible degradation) and to ensure accurate results. The thermal protocol applied during the DSC experiments is illustrated schematically in Figure 1. It consists of five sequential steps specifically designed to investigate the self-nucleation behavior in these TPU samples:

(a) Thermal history elimination: the sample was first heated to a suitable temperature, approximately 30 °C above its melting point, and held for 1 min to erase any prior thermal memory and generate an isotropic melt. The chosen temperatures were 250 °C for TPU29, TPU33, TPU50, and TPU60, 260 °C for TPU70 and 270 °C for TPU80.
(b) Standard semicrystalline morphology generation: the isotropic melt was cooled to 40 °C at a controlled rate of 20 °C/min to establish a reference semicrystalline structure.
(c) Self-nucleation step: the sample was reheated from 40 °C to a selected self-nucleation temperature (*T*_s_) and held there for 1 min. Although literature commonly uses a 5 min duration,^16–18,31^ a shorter time was intentionally chosen here to reduce the risk of thermal degradation.
(d) Controlled cooling phase: the sample was then cooled from *T*_s_ to 40 °C at the same rate of 20 °C/min. A 1 min stabilization period followed at 40 °C. After this step, the sample was collected for *ex situ* Wide-Angle X-ray diffraction analysis (WAXD, see next section).
(e) Final heating: last, the sample was recovered from the WAXD and heated from 40 °C to above its melting point at 20 °C/min.

**Figure 1:**
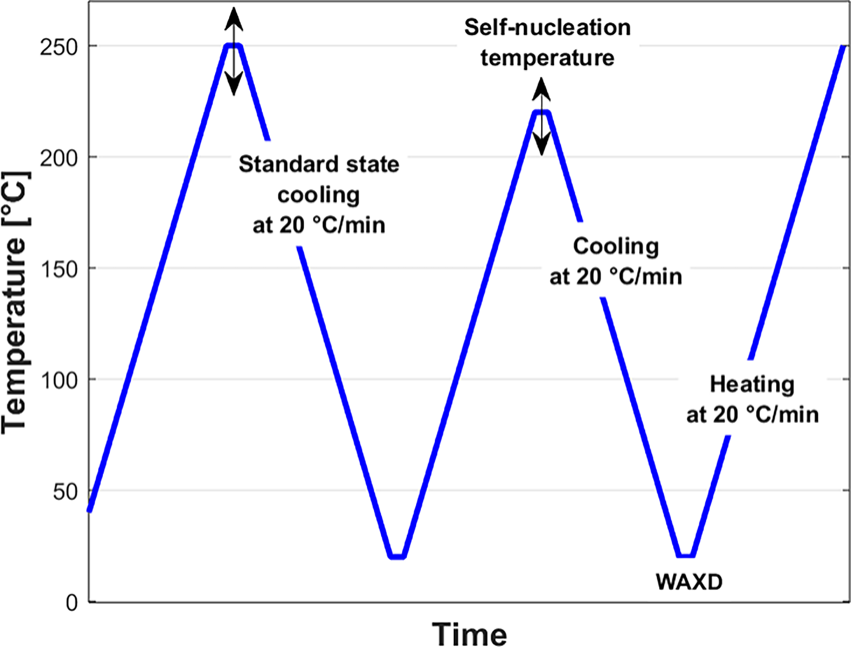
Schematic illustration of the self-nucleation protocol applied by DSC.

Thermal stability is crucial in analyzing MDI/BD-based urethanes and thermoplastic polyurethanes (TPUs). According to Yang et al.,^34^ noticeable degradation in MDI/BD urethanes begins at around 200 °C after exposure that exceeds 5 min. Additionally, solid-state degradation can occur during prolonged thermal treatments at high temperatures, such as after 2 h at 170 °C. Hwang et al.^35^ further demonstrated that signs of degradation can be detected via infrared spectroscopy when samples are maintained above 180 °C for over 5 min. This degradation process may involve simultaneous depolymerization and repolymerization, ultimately altering the molecular weight distribution.^34^

To limit these effects and ensure data reliability, fresh samples were prepared for each experiment (i.e., for each tested self-nucleation temperature), and isothermal exposure at elevated temperatures was restricted to 1 min. While the possibility of minor degradation cannot be entirely excluded, the applied thermal protocol and sample handling strategy significantly reduced the influence of degradation on the results.

### Wide-Angle X-ray Diffraction (WAXD)

WAXD measurements were performed using a MiniFlex diffractometer (Rigaku, Tokyo, Japan) equipped with a Cu K_α_ X-ray source (λ = 0.154 nm). The measurements were conducted in the θ*/2*θ scanning mode. One-dimensional (1D) scattering curves were obtained using SmartLab Studio software. The scanning parameters were set as follows: a start angle of 5° and an end angle of 40°, a step size of 0.05°, and a scanning speed of 2.5°/min. The X-ray generator was operated at a voltage of 40 kV and a current of 15 mA.

As mentioned above, sample preparation was performed in the DSC by cooling the samples from the selected self-nucleation temperature to 40 °C at a rate of 20 °C/min. Each sample for WAXD measurement weighed approximately 12 mg, and the polymers were manually removed from the aluminum pans before acquisition of the pattern.

### Temperature-Resolved Fourier Transform Infrared (FT-IR) Spectroscopy

To probe hydrogen bond’s dissociation upon heating to the self-nucleation temperature, in situ FT-IR measurements were carried out using a Nicolet Apex FT-IR spectrometer from Thermo Scientific coupled with a Linkam LNP96-S hot-stage for temperature control. Initially, a sample of 10 μm thickness was microtomed at room temperature to prevent the saturation of the absorbance. The sample was placed between the IR windows of the hot-stage and thermally conditioned to produce the standard semicrystalline state according to the above-described thermal history (first three steps of Figure 1). Eventually, the melt-crystallized sample was heated at a rate of 5 °C/min to 250 °C, while the FT-IR spectra were acquired using 32 scans, resulting in a temperature resolution of approximately one spectrum every 3.5 °C.

### Polarized Light Optical Microscopy (PLOM)

The samples’ microstructure was investigated using a polarized light optical microscope (Olympus BX53M) with a red-tint plate at 45° with respect to the polarizer and analyzer crossed position. The micrographs were taken with an Olympus SC50 camera. A Linkam THMS600 hot-stage, which was linked to a cooling device that employs liquid nitrogen, was used to apply the appropriate thermal procedure. Samples weighing approximately 30 mg were sectioned from the injection-molded sheet by using a sharp blade. Each sample was then placed between two microscope glass slides and compression-molded into a thin film of approximately 30 μm thickness. This was achieved by placing the glass slide assembly on a preheated hot plate set to the appropriate temperature and applying gentle pressure with tweezers to ensure uniform film formation. A self-nucleation thermal procedure similar to the one depicted in Figure 1 was applied to study the morphology of the samples after heating the TPU to the selected *T*_s_ temperature. In this case, the samples were heated for 3 min at a maximum temperature (instead of 1 min) to equilibrate the samples. Longer times than in the DSC were used due to the higher sample quantity employed in PLOM. Then, the sample was cooled down to 40 °C and heated to the selected *T*_s_ temperature. Finally, the material was cooled to 40 °C, and micrographs were recorded.

## Results and Discussion

### Differential Scanning Calorimetry

Figure 2 presents DSC scans obtained during the self-nucleation treatment of TPU with 60% and 80% HS content as representative examples. Figure 2a,c shows DSC cooling scans from the indicated *T*_s_ values and Figure 2b,d the subsequent heating scans. Results from various self-nucleation temperatures, *T*_s_, are presented. The literature typically uses red, blue, and green color codes to denote the different self-nucleation *Domains*, i.e., for *Domain I*, *Domain II*, and *Domain III*, respectively.^17,18^ In the present case, however, only the limit between *Domain I* and *Domain II* was clearly appreciable, while the *Domain II/Domain III* demarcation was uncertain (see the discussion later in the text). Therefore, we decided to use red curves to identify *Domain I* and a blue curve for the first *T*_s_ in *Domain II*, while the other curves at lower self-nucleation temperatures are drawn in black because of the impossibility of distinguishing between the measurements in *Domain II* and those in *Domain III*.

**Figure 2:**
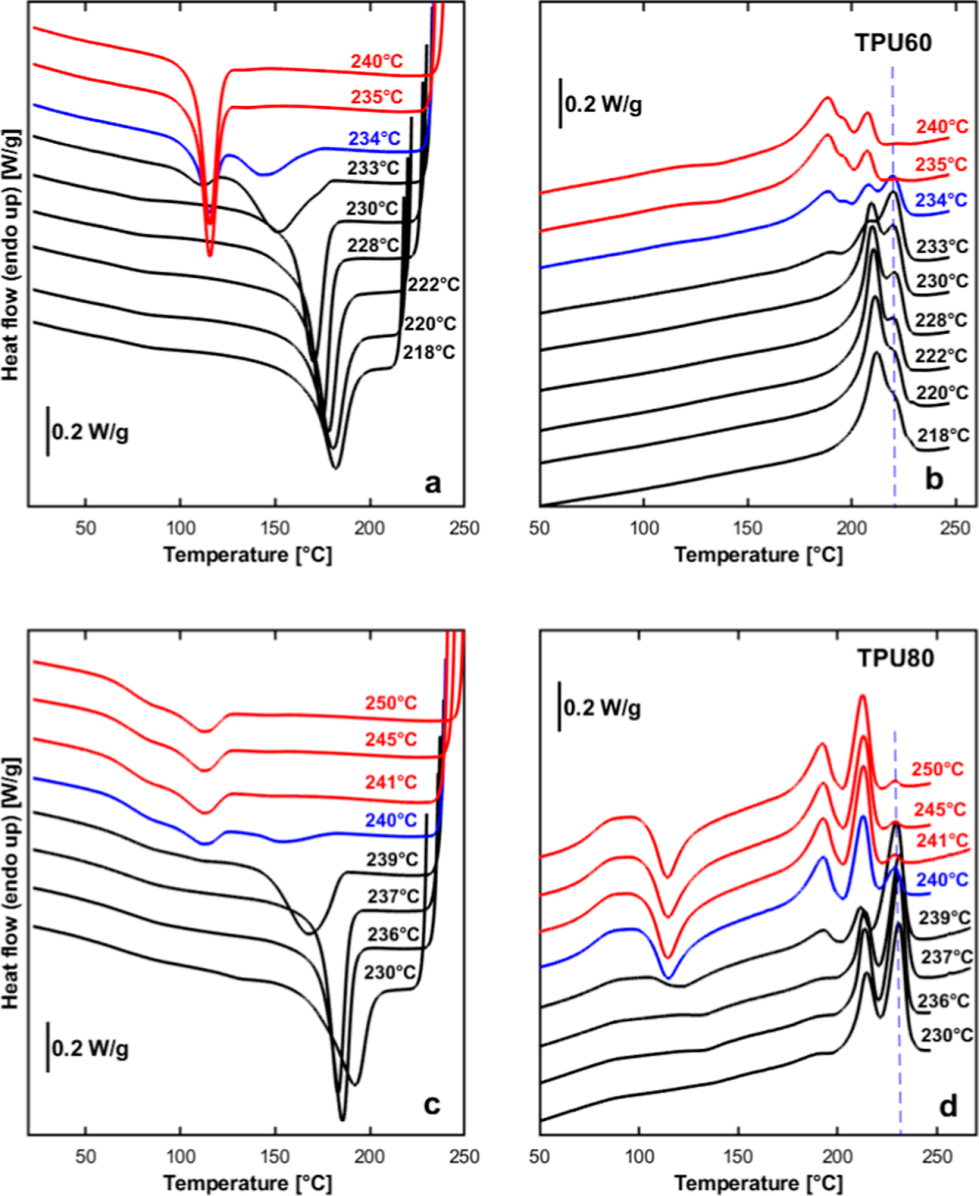
(a,c) Representative DSC scans of TPU 60% and TPU 80% during cooling at 20 °C/min from the indicated *T*_s_ values. (b,d) Subsequent DSC heating scans after the cooling scans shown in (a,c), also at 20 °C/min. The dashed vertical blue line indicates the melting temperature of *Form II*.

The DSC cooling curves in Figure 2 show a progressive change in the crystallization behavior of both TPUs with decreasing *T*_s_. At higher *T*_s_ values (e.g., in the 240–235 °C range for TPU60 and 250–241 °C for TPU80, respectively), a single low-temperature crystallization peak (at approximately 115 and 111 °C, for TPU60 and TPU80, respectively) is observed. Based on our previous work on the same materials,^14^ this peak is attributed to the crystallization of *Form I.* The fact that the peak crystallization temperature remains unchanged with varying *T*_s_ confirms that the samples are in *Domain I*, where self-nucleation does not influence the crystallization kinetics.

As *T*_s_ is decreased (to 234 and 233 °C for TPU60 and to 240 °C for TPU80), two crystallization exotherms are evident in the DSC cooling curves, with a second crystallization peak which emerges at higher temperatures (at approximately 140–150 °C) than the one assigned to *Form I.* This high-temperature crystallization exotherm gradually becomes more prominent, at the expense of the low-temperature one, as *T*_s_ is further reduced. According to the refs 11–13 and our own results,^14^ this high-temperature crystallization is due to the formation of *Form II*, a more ordered and thermodynamically stable polymorph. Given that the crystallization of this polymorph is induced by a decreased self-nucleation temperature and accompanied by a distinct increase in nonisothermal crystallization temperature, the related self-nucleation temperature region can be identified as *Domain II*. Notably, this emergence of double crystallization peaks upon entering *Domain II* was also reported in a previous work on TPU self-nucleation by Fernandez-D’Arlas et al., for TPU containing 30 and 43 wt % HS content.^31^ However, a previous work focused exclusively on the kinetics and morphology without discussing a possible change in the polymorphism of the materials with self-nucleation. The DSC heating scans in Figure 2 further support this interpretation, as they reveal distinct melting peaks corresponding to the two crystalline forms.

In most cases, when a polymer is self-nucleated, the crystallization temperature increases significantly, but its melting temperature either remains unchanged or increases very little. A typical example is isotactic polypropylene, for which an increase in *T*_c_ of 30 °C can be obtained while the melting temperature increases by a maximum of ∼2 °C.^16,36^

In contrast, the TPU samples studied here exhibit the appearance of a distinct melting endotherm at high temperatures upon decreasing the self-nucleation temperature from *Domain I* to *Domain II*. This extra melting peak, indicated with a vertical dashed blue line in Figure 2b,d, increases in importance with decreasing *T*_s_. Moreover, to corroborate its assignment to the more thermodynamically stable *Form II*, we note that it becomes evident only when the second high-temperature exotherm appears in the cooling scan (i.e., 233 °C for TPU60 and 240 °C for TPU80). As such, the materials’ thermal behavior (both on cooling and subsequent heating in Figure 2) supports the interpretation of a self-nucleation-induced polymorphic transition: from *Form I* crystallization, when cooling from high *T*_s_, to the predominance of *Form II* at lower self-nucleation temperatures.

Finally, the definition of *Domain III*, typically identified by the emergence of a small but clear “annealing peak” in the heating scans at temperatures higher than the main melting peak, could not be clearly established in any of the TPU systems. In fact, due to the typical polyurethane melting–recrystallization behavior,^12,37,38^ and to the onset and growth of *Form II* content with decreasing *T*_s_, it is not possible to unequivocally establish when *Domain II* ceases, and *Domain III* starts in the investigated self-nucleation temperature range and employed experimental conditions. Therefore, the DSC scans corresponding to this *T*_s_ range were plotted in black to indicate this uncertainty.

Although in different characteristic temperature regions, similar results were obtained for all of the investigated materials (see Figure S1). Notably, also the TPUs that did not develop *Form II* when cooling at 20 °C/min from a high-temperature melt (such as TPU29 and TPU33)^14^ were instead able to show the crystallization of this polymorph when self-nucleated in *Domain II* (Figure S1a,b).

Figure 3 shows the experimentally determined crystallization enthalpy of *Form I* and *Form II* as a function of self-nucleation temperature for the TPU samples with varying hard segment content. A general trend is observed across all samples: as *T*_s_ decreases, the enthalpy associated with *Form I* crystallization decreases from a plateau value to zero (or near zero). In contrast, the enthalpy of *Form II* increased over the same temperature range. This inverse trend of the enthalpies of the two structures reflects a gradual transition from *Form I* to *Form II* crystallization as the self-nucleation effect becomes more important (lower *T*_s_), inducing the formation of the more ordered polymorph (*Form II*).

**Figure 3:**
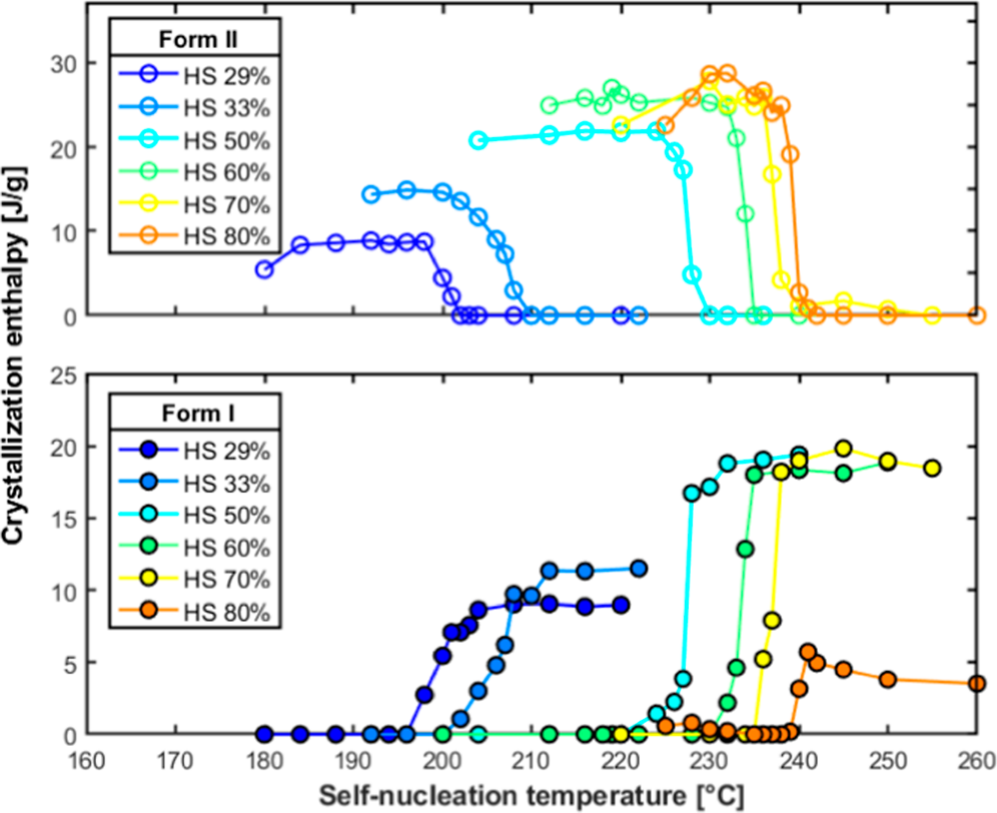
Crystallization enthalpy of *Form I* and *Form II* as a function of self-nucleation temperature for the different TPU samples employed in this work.

Significantly, the temperature range over which this transition occurs depends on the HS content. For instance, in TPU80, the switch from dominant *Form I* to *Form II* occurs at around 240 °C, whereas for TPU29, it takes place at a much lower temperature, around 200 °C. This result is linked to the different *Domain II* temperature intervals for the TPUs with varying HS content, which are associated with the various thermal stability of the self-nuclei, which in turn depend on that of the original crystals.

Additionally, the maximum crystallization enthalpy of each crystal form, which is indicative of the degree of crystallinity, is reported in Figure 4 against the HS content.

**Figure 4:**
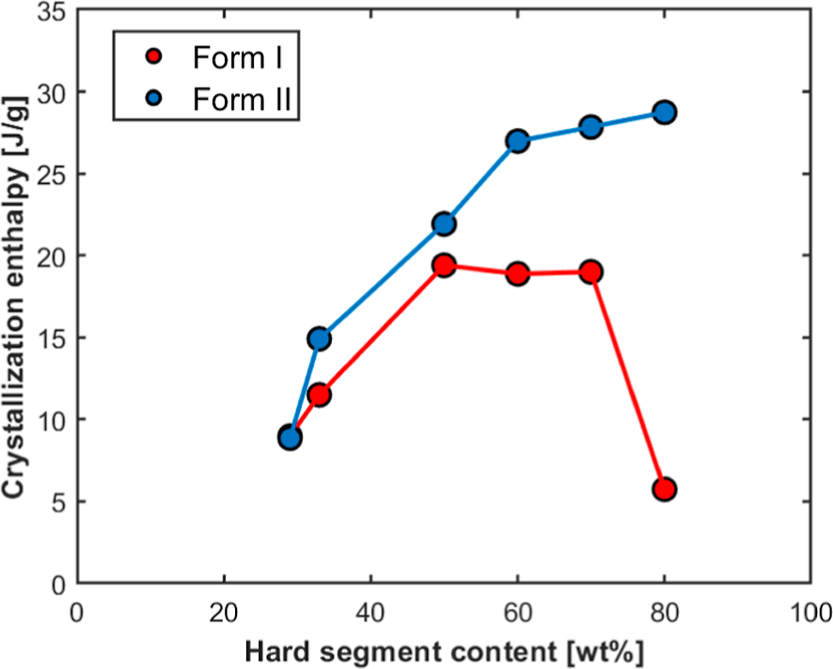
Maximum enthalpy of crystallization of *Form I* and *Form II* (at their respective ideal self-nucleation temperatures) versus hard segment content for the investigated TPUs.

It can be seen that the enthalpy of *Form II* increases monotonically with increasing HS content, starting from below 10 J/g and reaching a plateau at around 30 J/g. In contrast, the enthalpy of *Form I* exhibits a nonmonotonic trend: it increases to a plateau slightly below 20 J/g (lower than that of *Form II*) for HS contents between 50 and 70 wt % and then decreases to approximately 5 J/g for TPU80. Interestingly, TPU80 exhibits the lowest *Form I* enthalpy, which is somewhat counterintuitive given its high HS content. This decrease clearly indicates a more hindered crystallization of TPU80 with respect to the other samples and can be attributed to its low crystallization temperature in *Domain I*, which is close to the polymer’s glass transition temperature,^14^ thereby restricting chain mobility and limiting crystallization, as it will be discussed in more detail further on. For *Form I*, the drop in enthalpy for TPU33 and TPU29 is linked to the lower content of crystallizable HS and to the declining capacity of these systems to organize into the *Form I* structure.

In contrast, the crystallization enthalpy of *Form II* is comparable among TPUs with higher HS content (TPU80, TPU70, TPU60, and TPU50). This result indicates that once the self-nucleation-induced transition to *Form II* occurs, crystallization becomes more efficient for TPU80 due to its significantly higher crystallization temperature, which is well above the glass transition temperature. This eliminates the diffusion limitations observed in *Domain I*. On the other hand, the enthalpy of *Form II* crystallization is lower for TPU33 and TPU29 due to the smaller content of hard segments in the polymer.

Figure 5 illustrates the evolution of the crystallization temperatures for *Form I* and *Form II* as a function of the self-nucleation temperature across all TPU samples with varying hard segment content. At high *T*_s_ values, a plateau in the crystallization temperature (*T*_c_) is observed for *Form I*, indicating that the crystallization of this polymorph is unaffected by the self-nucleation temperature in *Domain I*, as expected. This plateau is only slightly dependent on the HS content: while TPU29 and TPU33 exhibit somewhat lower *T*_c_ values, the samples with 50–80% HS content show similar crystallization temperatures, suggesting comparable nonisothermal crystallization kinetics among these higher-HS materials.

**Figure 5:**
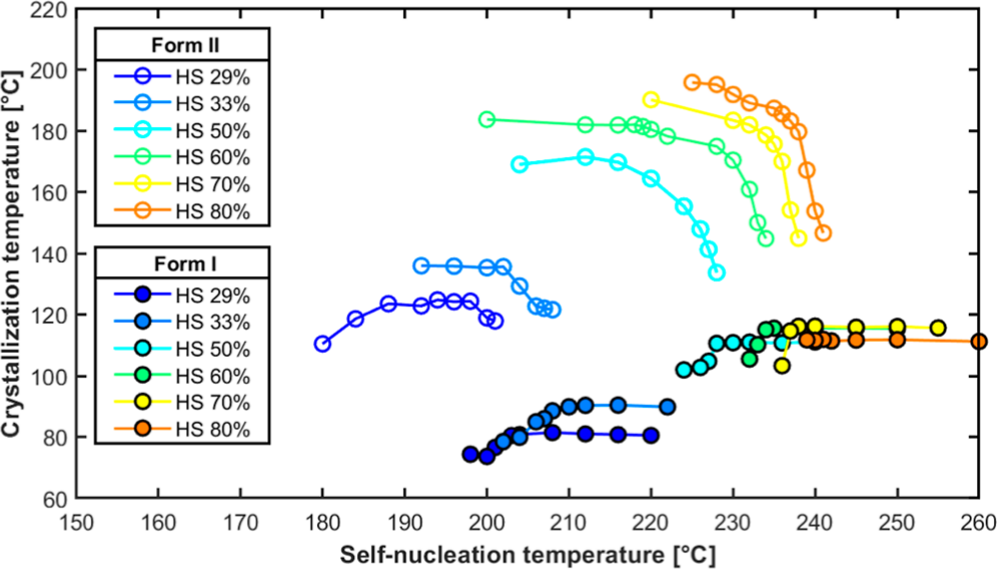
Crystallization temperatures of *Form I* and *Form II* as a function of self-nucleation temperature for the different TPU samples.

Figure 5 also shows that as *T*_s_ decreases, a second, higher-temperature crystallization peak associated with *Form II* begins to appear. This result indicates the onset of a polymorphic transition that is induced by self-nucleation. There is a range of *T*_s_ values where both crystallization peaks coexist, consistent with observations in DSC analysis (e.g., Figure 2, *T*_s_ values of 234–233 and 240 °C for TPU60 and TPU80, respectively). This self-nucleation region corresponds to a regime in which *Form II* nucleates and crystallizes first to a certain extent, followed by the development of *Form I* at lower temperatures during the same cooling run.

Importantly, while the *T*_c_ of *Form I* remains largely unchanged with decreasing *T*_s_ (at high self-nucleation temperatures), the *T*_c_ of *Form II* increases progressively until it reaches a plateau. This trend indicates that self-nucleation not only facilitates a transition from *Form I* to *Form II* crystallization but also enhances the nucleation density of *Form II*, thereby raising its crystallization temperature. The rise in the *T*_c_ of *Form II* with self-nucleation is more significant at a higher HS content. For example, the overall increase in crystallization temperature (from the plateau values exhibited by *Form I* in *Domain I* to the plateau values for *Form II* at low *T*_s_) ranges from about 44 °C for TPU29 to nearly 85 °C for TPU80. These values align closely with those reported in the literature for TPU with 33 and 40 wt % HS.^31^ At very low *T*_s_ values, a slight decrease in the *T*_c_ of *Form I* is observed. This subtle drop may be due to spatial confinement effects, in which *Form I* crystallization is restricted within the preformed scaffold of *Form II* spherulites (see later in the PLOM section) that nucleate and grow earlier at higher temperatures. Such structural confinement may hinder the ideal growth of *Form I* crystals, slightly depressing their crystallization kinetics.

To better understand the kinetics of polymorphs’ formation in relation to HS content, the maximum reached crystallization temperatures of the two structures (at the respective ideal self-nucleation temperature) are plotted as a function of HS weight percent in Figure 6. The glass transition temperature, measured via fast scanning calorimetry in a previous publication,^14^ is also reported.

**Figure 6:**
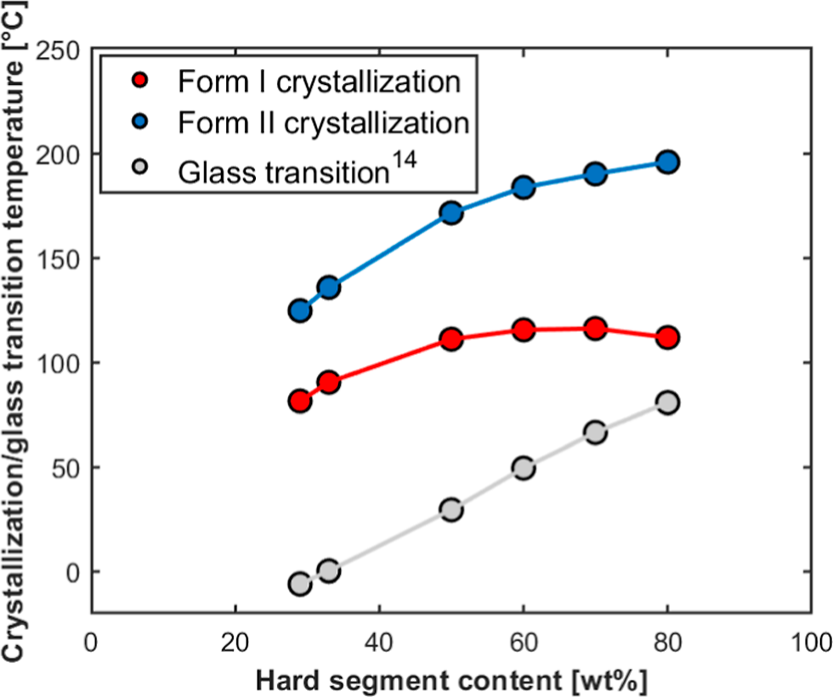
Polymorphs’ crystallization and polymer glass transition temperatures as a function of HS content for the investigated TPU samples.

Considering *Form I*, the crystallization temperatures are similar for HS contents between 50 and 80 wt %. However, the polymers’ glass transition temperature, measured in a previous work,^14^ changes with composition. Therefore, while *Form II* crystallization occurs for all the samples well above the polymer’s glass transition temperature, the distance between *Form I* crystallization temperature and the glass transition becomes narrower with increasing HS content. Therefore, the absolute crystallization temperature alone is not the sole factor influencing the kinetics of *Form I* development; additionally, the proximity to the glass transition temperature also plays a critical role. In fact, at the highest HS content, *Form I* crystallization temperature (about 112 °C) is relatively close to the sample’s glass transition (roughly 81 °C), or at least closer than in TPU70 (*T*_C_ = 116 °C and *T*_g_ = 66 °C). This corroborates the suggestion of diffusion limitations for the crystallization of *Form I* in TPU80.

Room-temperature WAXD was cooled from different T_s_ temperatures.

Figure 7a,b displays representative WAXS patterns of TPU60 and TPU80, collected at room temperature after cooling from selected *T*_s_. For the sake of comparison, a pattern of the melt at 250 °C collected at the synchrotron^14^ and shifted on the *2*θ axis for clarity is also added. For TPU60, at higher *T*_s_ values (240 and 235 °C), the diffractograms are dominated by a small diffraction peak centered around 2θ ≈ 20.5°, superimposed on a broad amorphous halo. Moreover, a weak diffraction peak is discernible at around 2θ ≈ 12°, indicated by an arrow. The shape of these diffraction patterns is compatible with the crystallization of the paracrystalline *Form I*, which is known to exhibit low-intensity diffraction due to its loose chain packing and low degree of structural order.^12^ A similar pattern is observed for TPU80 at a *T*_s_ of 240 °C, while at higher self-nucleation temperatures, the amorphous component dominates for this polymer due to the low crystallinity, and only traces of *Form I* may be present, in agreement with the DSC scans of Figure 2c,d, which show a small crystallization exotherm and strong cold-crystallization upon subsequent heating (see also the low *Form I* crystallization enthalpy displayed in Figure 3).

**Figure 7:**
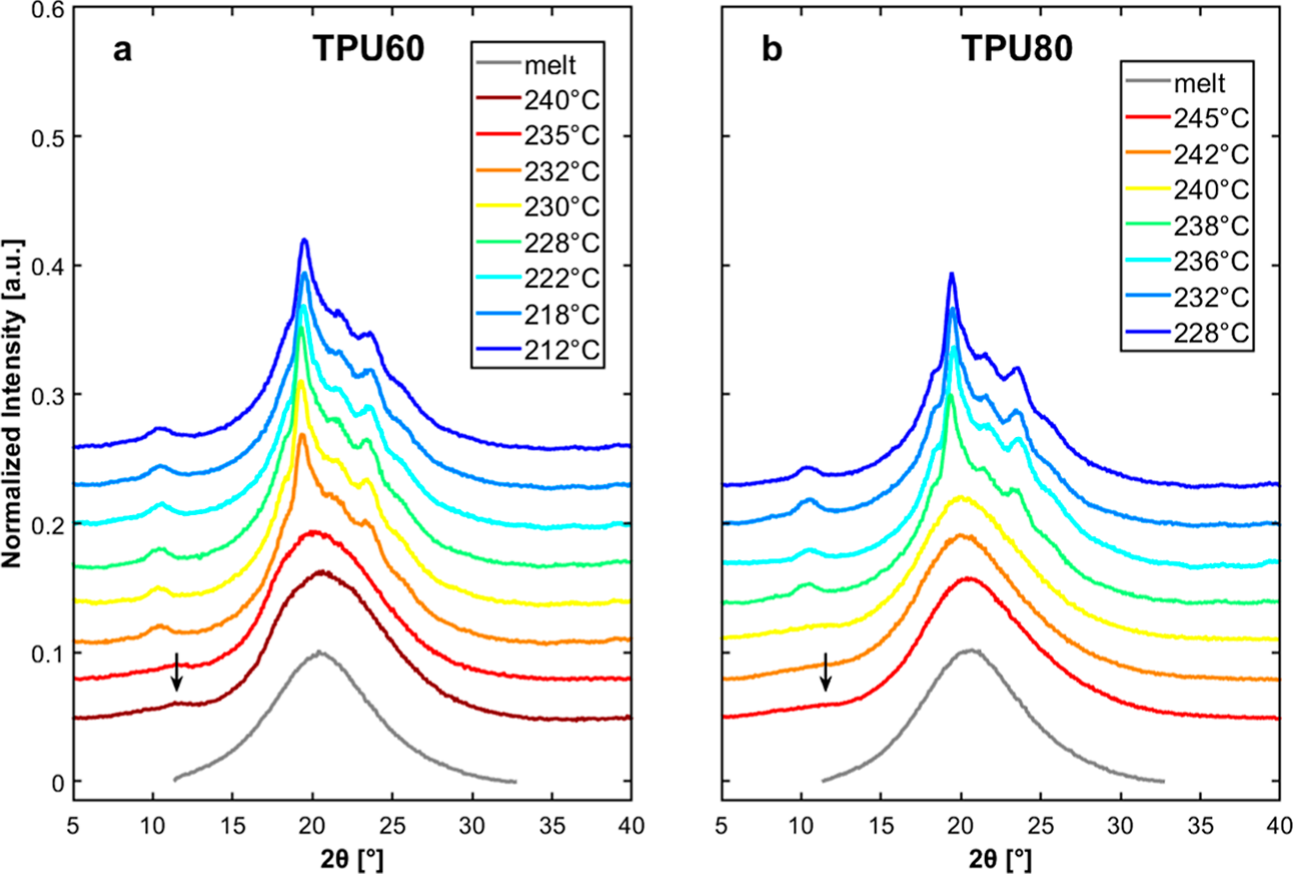
Room-temperature WAXD patterns for TPU60 (a) and TPU80 (b) after cooling the samples from the indicated self-nucleation temperatures. The patterns of the molten sample^14^ (corrected for temperature and wavelength) are also reported, for the sake of comparison.

As the self-nucleation temperature decreases and enters *Domain II*, within the respective ranges for TPU60 and TPU80, the patterns exhibit a significant change, marked by the emergence of several crystalline diffraction peaks (see *T*_s_ 232 and 238 °C for TPU60 and TPU80, respectively). In particular, the small peak at around 2θ ≈ 10° and the strong diffraction at around 2θ ≈ 20° are easily recognizable. According to the literature, these reflections correspond to the (004) and (104) planes of the crystalline *Form II*.^11,33^ *Form II* diffraction peaks progressively increase in intensity as *T*_s_ is reduced, suggesting a gradual increase in *Form II* content at the expense of *Form I* induced by self-nucleation, in line with what is observed from the crystallization enthalpies of the two forms in Figure 3. An analogous behavior was observed for all of the samples with varying HS content (see Figure S2), with a noticeable decrease in the intensity of *Form II* diffraction for the polymers containing less hard segments compared to the more rigid ones.

To quantitatively describe the observed trend, the maximum intensity of the (104) peak of *Form II* has been evaluated for different *T*_s_ values. The results for the various TPUs are reported in Figure 8. The analysis steps involved a linear baseline subtraction from 5° to 40° of 2θ, followed by normalizing the area of each pattern to the respective integrated value in the same angular range. In this way, every resulting normalized pattern has an area equal to unity. From these diffractograms, the maximum intensity in the angular range of 18.8–20.8° of 2θ is identified, specifically in the region of the emergence of the *Form II* (104) peak.

**Figure 8:**
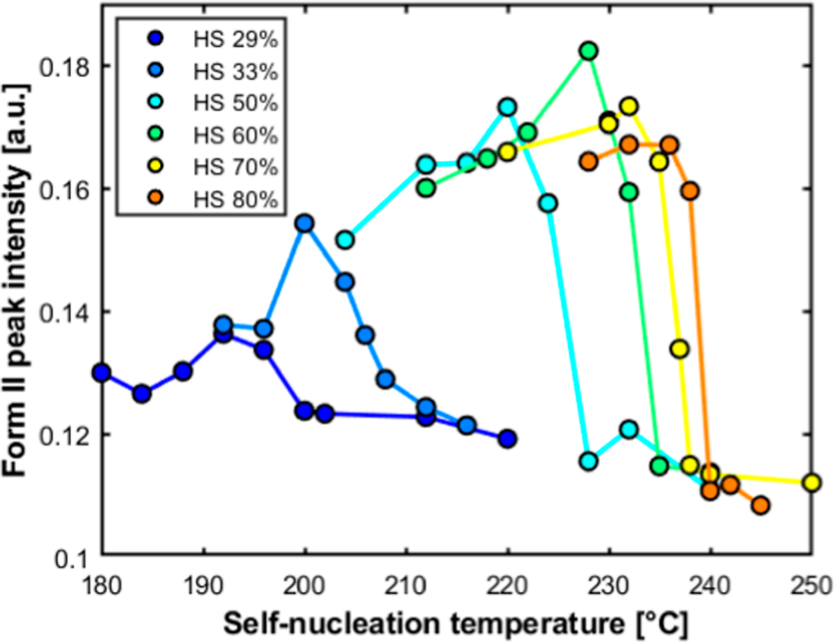
Trend of measured *Form II* peak intensity as a function of self-nucleation temperature for the different TPU samples with varying HS content.

While a deconvolution of the patterns is theoretically possible, it is essential to note that our samples typically consist of three phases: *Form I*, *Form II*, and the amorphous phase. *Form II* alone can exhibit up to six distinguishable crystalline peaks, while *Form I* contributes two peaks. Additionally, all samples exhibit a broad but significant amorphous halo. Consequently, deconvoluting each pattern would require fitting contributions from at least nine peaks. Given the potential variability in selecting peak shapes and fitting functions, we believe that such a deconvolution would be quite arbitrary and heavily dependent on the specific fitting parameters. This is especially true for samples rich in *Form I*, where the diffraction pattern is relatively featureless, making reliable deconvolution even more challenging.

The data in Figure 8 confirm quantitatively the trend observed in Figures 7 and S2. In particular, the extracted *Form II* peak intensity for each sample has approximately a low plateau value at high self-nucleation temperatures before increasing to a maximum point upon decreasing the *T*_s_. Notably, the rise in *Form II* content parallels the appearance of the high-crystallization temperature peak in the DSC scans in terms of temperature intervals (see Figures 2 and 3). The onset of the *Form II* crystallization temperature, which also corresponds to the *Domain I/Domain II* boundary, shows a clear dependence on the HS content, decreasing from approximately 240 °C for TPU80 to about 200 °C for TPU29. This decrease is possibly linked to the analogous shift of melting temperatures with hard segment content. It is worth noting that the *Form II* peak intensity appears to show a maximum with *T*_s_. The decreasing part of the curves at low self-nucleation temperatures, when *T*_s_ enters the melting endotherm, could tentatively be attributed to the partial melting of the original *Form I* crystals, the standard state before self-nucleation. After this partial melting, only the unmolten part of the material recrystallizes into *Form II*, reducing its final content in the sample alongside the remaining *Form I* crystals.

Both the analyses of DSC (Figure 4) and WAXD (Figure 8) data confirm the promotion of *Form II* crystallization by self-nucleation. Interestingly, the crystallization of this polymorph is triggered despite the fact that the original crystals present before the self-nucleation procedure (i.e., the standard state created at the beginning; see Figure 1) consist of the other polymorph, the paracrystalline *Form I.* Thus, self-nucleation induces a change in the recrystallizing polymorph for temperatures in *Domain II*.

While melt memory is often known to control the polymorphic outcome of polymer crystallization, the specific trend reported in this work is rather peculiar. In fact, in most cases, when starting from a given polymorph as the standard state, the self-nuclei of this original structure often need to be erased at high temperatures (corresponding to *Domain I*) to promote the recrystallization of the other structure. This is, for instance, the case of syndiotactic polystyrene,^19,20^ for which the melting kinetics of α-phase nuclei has even been derived,^20^ and of isotactic poly­(1-butene),^21,22^ where the kinetically favored *Form II* can only develop when the memory of the thermodynamically most stable *Form I* has been erased. Similar examples concern the polymorphic crystallization of the polyesters poly­(3-hydroxypropionate)^24^ or poly­(butylene adipate).^25^ On the other hand, in a few cases, self-nucleation in temperatures within *Domain II* causes the formation of a polymorph that is different from the one present in the standard state, the most studied case being that of β-phase isotactic polypropylene, which recrystallizes in the α-phase.^26,27^ Analogous behavior is displayed by poly­(pivalolactone),^28^ which can form γ-phase crystals starting from the α-structure under certain melting and crystallization conditions. This behavior is rationalized by proposing that the survival of a structural organization characteristic of the second modification in the melt is necessary for the proper self-nucleation conditions. It should be noted that in the two cases described above, similar to the TPUs, the one corresponding to isotactic polypropylene is actually really analogous because the self-nucleation of a metastable structure (β-phase) induces the stable one (α-phase).^26,27^ For poly­(pivalolactone), instead, the thermodynamic stability of the two polymorphs is the opposite, i.e., self-nucleation of the stable polymorph leads to the metastable one.^28^

A reason behind this structural change in TPUs induced by self-nucleation should be proposed. At first, we recall that in their investigation on the polymorphism of MDI/BD-based polyurethanes analogous to those employed in this work, Wang et al.^11^ proposed that the amount of urethane groups involved in hydrogen bonds (i.e., the fraction of C=O groups hydrogen-bonded with N–H groups) is larger for *Form II* with respect to *Form I*. Moreover, in situ Fourier transform infrared spectroscopy during melting a TPU sample crystallized into *Form I* revealed that a substantial amount of hydrogen-bonded C=O groups persists above the DSC measured melting temperature.^39^ In fact, the dissociation of the interurethane hydrogen bonds with temperature is gradual, leading to a “heterogeneous” melt containing residual uncorrelated hard domains, as previously suggested also by small-angle X-ray scattering measurements.^40^

To confirm this conclusion from the literature, temperature-resolved FT-IR measurements were carried out on a selected sample (TPU60) upon heating after the sample was crystallized in the standard state according to the thermal protocol employed for DSC measurements (Figure 1). Figure 9 reports some of the acquired spectra, focusing on the carbonyl group stretching region (around 1700 cm^–1^). A clear splitting of the absorption band can be noticed. In particular, two different bands are found at about 1704 and 1734 cm^–1^, corresponding to the hydrogen-bonded and free C=O, respectively.^39,41^

**Figure 9:**
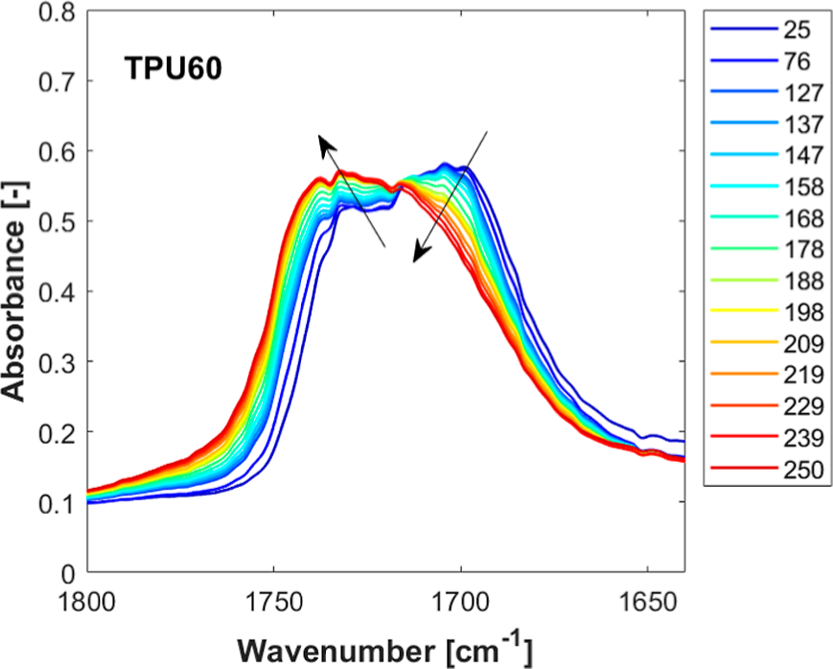
Temperature-resolved FT-IR spectra in the carbonyl stretching region for TPU60 crystallized from the melt in the standard state and subsequently heated at 5 °C/min. The arrows indicate the changes in the absorbance of the free and hydrogen-bonded carbonyls (left to right, respectively).

Upon heating of the sample, a clear gradual decrease in the absorbance of the 1704 cm^–1^ band and a simultaneous increase in the band around 1734 cm^–1^ can be identified. This indicates a progressive dissociation of the hydrogen-bonded carbonyl groups with increasing temperature. Interestingly, a certain small fraction of hydrogen bonds seems to persist in the TPU melt, i.e., above 229 °C. However, the sensitivity of the FT-IR technique does not allow us to clearly distinguish the decreasing trend in the self-nucleation temperature region (234–228 °C), possibly due to the very low content of hydrogen bonds at high temperatures. This notwithstanding, it is reasonable to conclude that an analogous temperature dependence to the one observed in the melting process should also apply to the higher temperatures.

Since hydrogen bonds are known to play an essential role in the self-nucleation of a different class of polymers, i.e., polyamides,^42^ by analogy, we can put forward that also in the case of thermoplastic polyurethanes, increasing the concentration of hydrogen bonds (by lowering self-nucleation temperature) will substantially accelerate the crystallization kinetics due to an increased number of nucleation sites for the HS. Moreover, we suggest that given the larger hydrogen-bonded urethane groups fraction in *Form II* with respect to *Form I*,^11^ an increased content of C=O/N–H hydrogen bonds with lowering *T*_s_ ^39^ will naturally promote the formation of the stable polymorph (*Form II*), although the TPU was originally crystallized in the metastable *Form I*.

Figure 10 presents the PLOM micrographs of TPU samples with varying HS content after cooling from different self-nucleation temperatures. The images clearly illustrate the combined effects of *T*_s_ and HS content on the semicrystalline morphology and nucleation density. The selected self-nucleation temperatures are representative of conditions under which, according to the DSC and WAXD data, one should have a predominance of *Form I* (higher *T*_s_), a majority of *Form II* (lower *T*_s_), and a mixture of the two polymorphs (intermediate *T*_s_).

**Figure 10:**
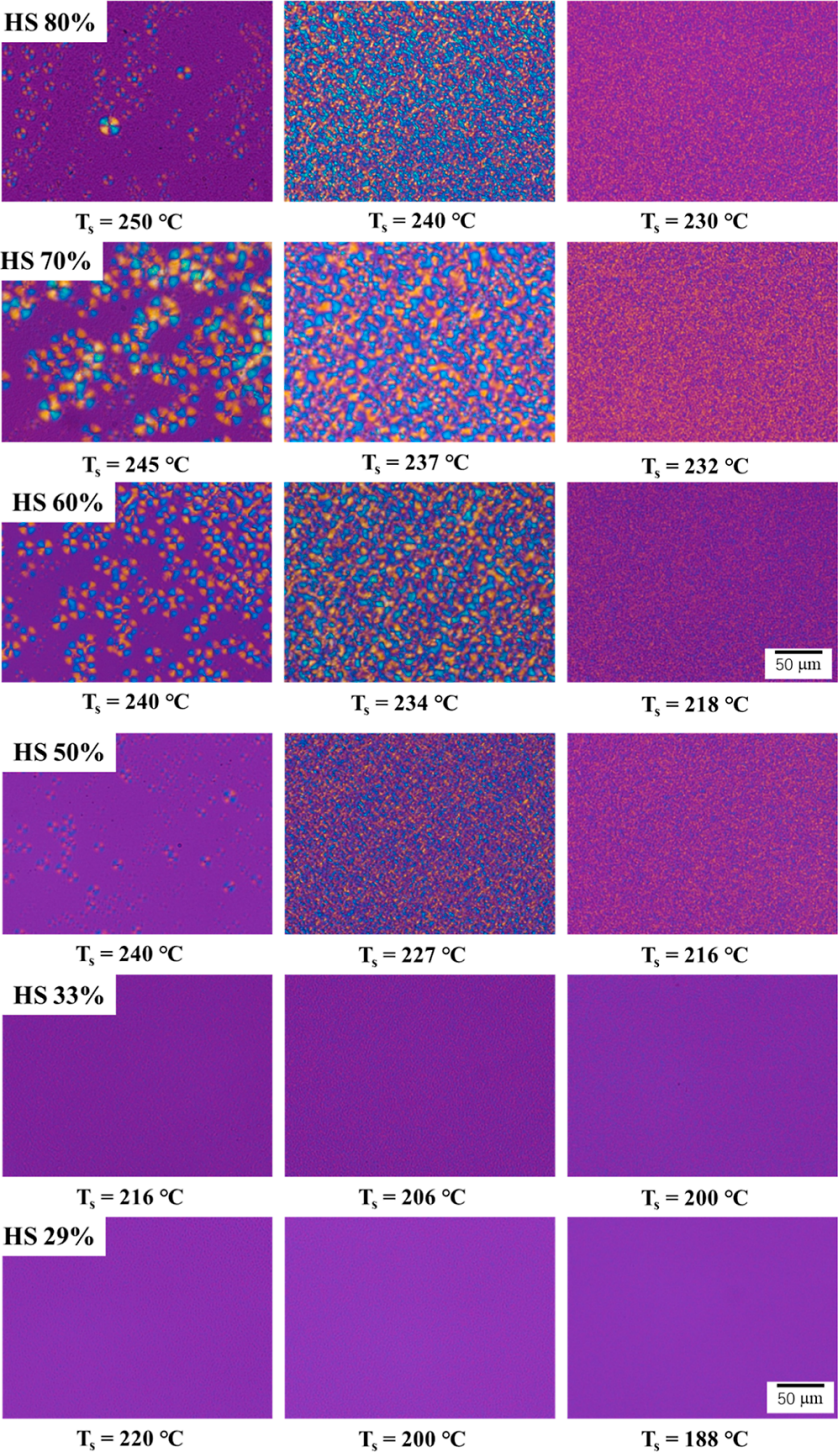
PLOM micrographs at room temperature acquired after cooling TPU with varying hard segment content from different *T*_s_.

Based on the literature^11,13^ and of our previous work,^14^ the two structures should have distinct morphologies, with *Form II* giving rise to birefringent spherulites characterized by well-distinguishable Maltese crosses. At the same time, *Form I* is featureless and nonbirefringent. As such, the relative content of *Form II* may be estimated by the fractional area of the micrograph covered by the birefringent spherulites. At high self-nucleation temperatures, the nonbirefringent area of the micrograph predominates for TPU29, TPU33, TPU50, and TPU80, indicating that *Form I* is present in higher amounts with respect to *Form II*. On the other hand, a considerable amount of birefringent spherulites (*Form II*) is present, even at the highest employed *T*_s_, for TPU60 and TPU70, suggesting a mixed polymorphic composition for these samples. The apparent difference between PLOM and DSC outcomes may be attributed to the different sample sizes and thus precise thermal histories; however, it is not particularly meaningful.

For TPUs with HS content equal to or above 50 wt %, a clear trend emerges: as *T*_s_ decreases to the chosen intermediate value, the nucleation density increases, with a larger number of birefringent crystalline domains seen under polarized light, which covers most of the observed area. This reflects the enhanced formation of *Form II*. In TPU80, for instance, only a few birefringent regions are visible at higher *T*_s_ (250 °C), while a much denser microspherulitic morphology appears at lower *T*_s_ (240 °C), indicating enhanced nucleation and *Form I*-to-*Form II* transition in *Domain II*. Similar nucleation behavior is evident in TPU70, TPU60, and TPU50, although the overall birefringence intensity diminishes slightly with decreasing HS content (more evident in TPU50), possibly due to the lower overall crystallinity.

In contrast, TPU33 and TPU29, which have low HS contents, show no detectable birefringent spherulites across all of the explored *T*_s_ values. The optical isotropy under PLOM at high and intermediate *T*_s_ implies the absence or minimal presence of *Form II*, which, according to DSC and WAXD measurements (Figures 2, 4, and 7), should instead be present at the lowest investigated *T*_s_. However, the small amount of HS may prevent the formation of distinguishable birefringent spherulites.

Some considerations can be made regarding the final properties of the materials, given that self-nucleation allows tailoring of the polymorphic content. Both theoretical estimations^11^ and experimental measurements^43^ indicate that *Form II* exhibits a higher elastic modulus compared to *Form I*. Therefore, applying low self-nucleation temperatures to the TPUs would enhance the stiffness of the resulting material—at a given cooling rate during processing—by increasing the content of the thermodynamically more stable *Form II*. Furthermore, it has been shown that the two polymorphs lead to different foamed structures.^44^ Specifically, samples subjected to self-nucleation and thus enriched in *Form II* would produce TPU foams with significantly lower cell density compared to samples crystallized primarily in *Form I*.

## Conclusions

Thermoplastic polyurethanes with hard segment contents ranging from 29 to 80 wt % have been studied, focusing on their self-nucleation behavior. Differential scanning calorimetry measurements revealed a single low-temperature peak when the sample is cooled from relatively high temperatures in the so-called *Domain I* (isotropic melt). On the other hand, when the self-nucleation *Domain*, i.e., *Domain II*, is entered, a second high-temperature peak appears next to the previous one. This high-temperature crystallization event gradually increases in enthalpy at the expense of the low-temperature peak with a decreasing self-nucleation temperature until it becomes the only peak.

The DSC findings are interpreted in light of the known polymorphism of TPUs: high self-nucleation temperatures induce the crystallization of the polymers in the metastable *Form I* (low-temperature peak), which is gradually replaced by the formation of the more stable *Form II* as *T*_s_ decreases. Hence, self-nucleation, in addition to causing the expected increase in crystallization kinetics, also promotes the formation of a different polymorph. Notably, the polymorph favored by self-nucleation is distinct from the one produced during the initial cooling at the same rate, i.e., the standard state before self-nucleation. Ex situ wide-angle X-ray diffraction measurements corroborate this interpretation on samples cooled from different *T*_s_, which confirms the promotion of *Form II* at the expense of *Form I* through self-nucleation. Polarized light optical microscopy measurements carried out at room temperature after cooling the samples from various self-nucleation temperatures support this scheme. Indeed, birefringent spherulites of *Form II* become more numerous and occupy a larger fractional area as the self-nucleation temperature decreases.

Self-nucleation-induced polymorphic crystallization is consistently found across the entire range of TPU compositions studied, with the HS content determining only the specific temperature interval for the phenomenon to occur. Interestingly, even TPUs with low HS content (i.e., 29% and 33%), which previous studies have shown are unable to produce *Form II* during nonisothermal crystallization, can develop this polymorph with the aid of self-nucleation.

The self-nucleation-induced formation of *Form II*, originating from *Form I* crystals in polyurethanes, is attributed to the persistence of interurethane hydrogen bonds in the melt. Indeed, since *Form II* is suggested to have a higher fraction of bonded C=O/N–H groups compared to *Form I*, a greater content of hydrogen bonds maintained at lower self-nucleation temperatures could favor the crystallization of the thermodynamically more stable *Form II* structure.

## Supplementary Material



## Notes

DSC cooling and heating scans form different T_S_ for other TPUs and room-temperature WAXD patterns after cooling from different T_S_ for other TPUs (PDF)
