# Supplementary material for: Self-Nucleation Enables Polymorphic Selection in Thermoplastic Polyurethanes
Source: Macromolecules. 2025 Sep 12;58(18):9876–86. doi: 10.1021/acs.macromol.5c01477 (PMC12462247; doi:10.1021/acs.macromol.5c01477)
Supplement: Supplementary file 1 [file ma5c01477_si_001.pdf]

Self-nucleation enables polymorphic selection in thermoplastic polyurethanes

Zakarya Baouch[1], Leire Sangroniz[2], Yunxiang Shi[2], Elmar Poeselt[3], Alejandro J. Müller[2,4]*, Dario Cavallo[1]*

[1] *Department of Chemistry and Industrial Chemistry, University of Genoa, Via Dodecaneso 31, 16146, Genoa (Italy)*

[2] *POLYMAT and Department of Polymers and Advanced Materials: Physics, Chemistry and Technology, Faculty of Chemistry, University of the Basque Country UPV/EHU, Paseo Manuel de Lardizábal, 3, Donostia-San Sebastián 20018, Spain*

[3] *BASF, Polyurethanes GmbH, Elastogranstrasse 60, Lemförde, Germany*

[4]*IKERBASQUE, Basque Foundation for Science, Plaza Euskadi 5, 48009, Bilbao, Spain*

SUPPORTING INFORMATION

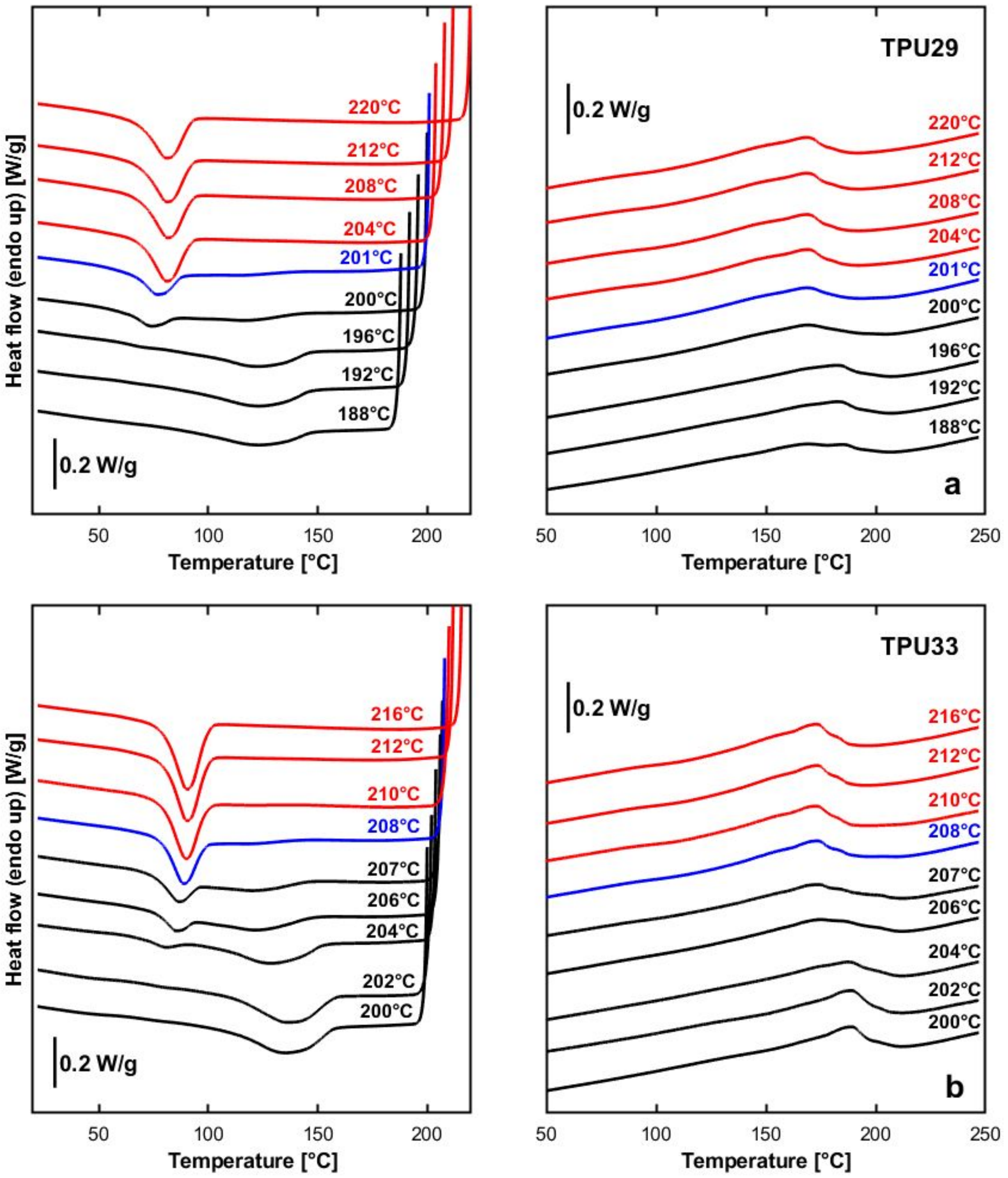

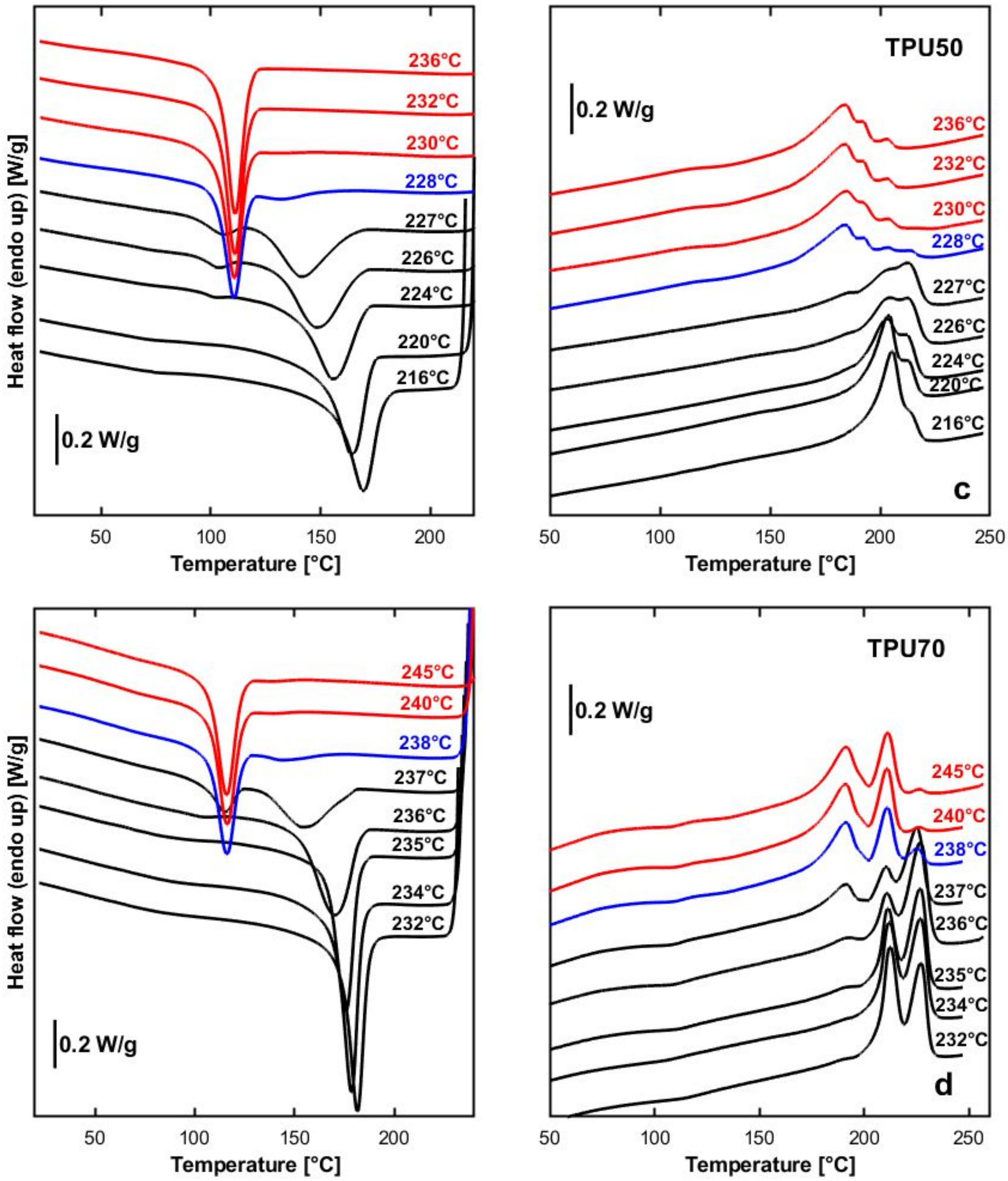

Figure S1: DSC cooling (left) and heating (right) scans at different T_s for TPU29 (a), TPU33 (b), TPU50 (c), TPU70 (d).

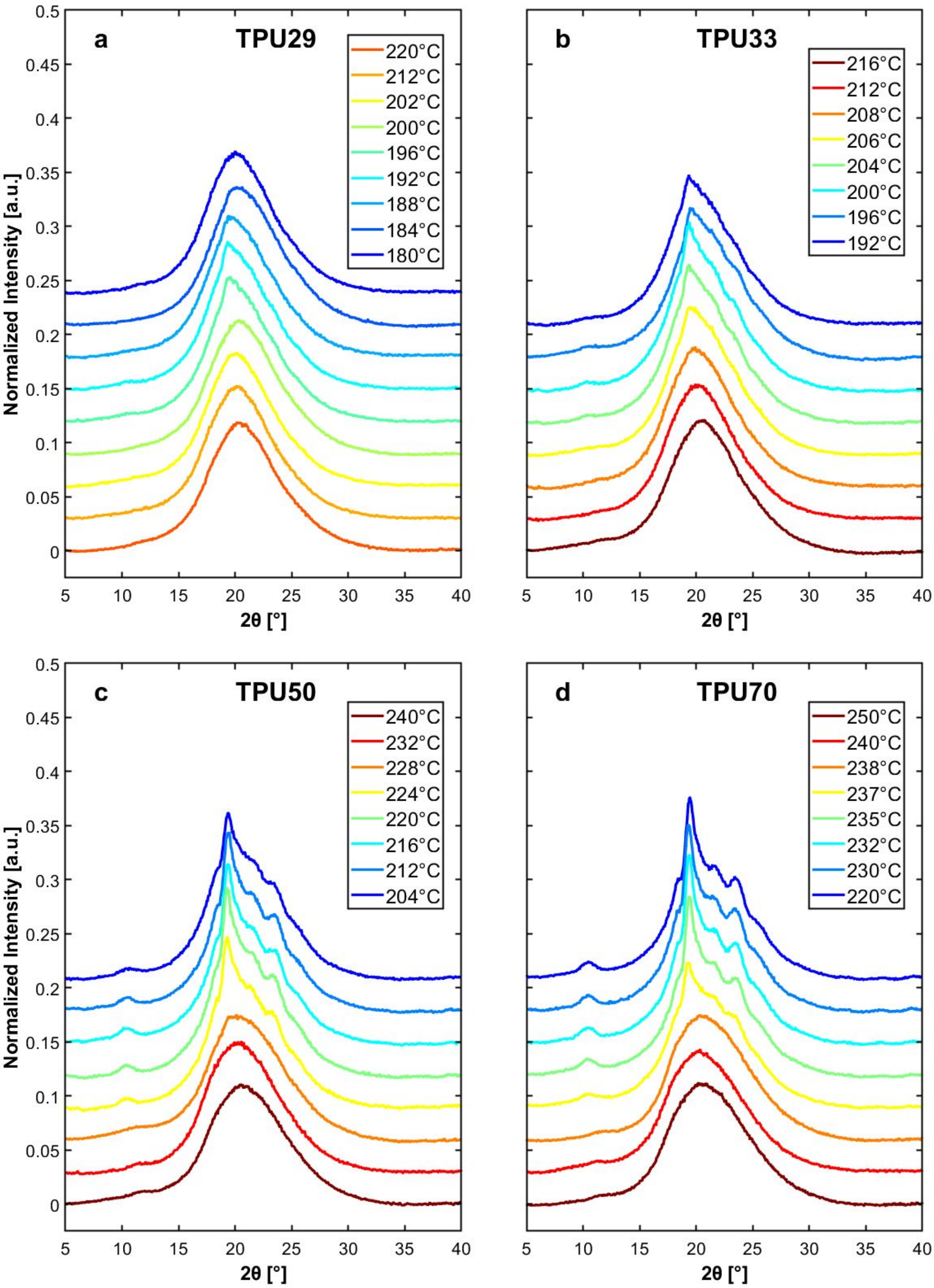

Figure S2: WAXD patterns for TPU29 (a), TPU33 (b), TPU50 (c), TPU70 (d), acquired after cooling the samples at 20 °C/min from different T_s.
